# Modified Glasgow Prognostic Score is predictive of prognosis for non-small cell lung cancer patients treated with stereotactic body radiation therapy: a retrospective study

**DOI:** 10.1093/jrr/rrab021

**Published:** 2021-04-19

**Authors:** Zhe Chen, Hotaka Nonaka, Hiroshi Onishi, Eiji Nakatani, Yoko Sato, Satoshi Funayama, Hiroaki Watanabe, Takafumi Komiyama, Kengo Kuriyama, Kan Marino, Shinichi Aoki, Masayuki Araya, Licht Tominaga, Ryo Saito, Yoshiyasu Maehata, Mitsuhiko Oguri, Masahide Saito

**Affiliations:** Department of Radiology, School of Medicine, University of Yamanashi, Chuo, Yamanashi, 409-3898, Japan; Department of Radiology, Shizuoka General Hospital, Shizuoka, Shizuoka, 420-8527, Japan; Department of Radiology, Fuji City General Hospital, Fuji, Shizuoka, 417-8567, Japan; Department of Radiology, School of Medicine, University of Yamanashi, Chuo, Yamanashi, 409-3898, Japan; Division of Statistical Analysis, Research Support Center, Shizuoka General Hospital, Shizuoka, Shizuoka, 420-8527, Japan; Division of Statistical Analysis, Research Support Center, Shizuoka General Hospital, Shizuoka, Shizuoka, 420-8527, Japan; Department of Radiology, School of Medicine, University of Yamanashi, Chuo, Yamanashi, 409-3898, Japan; Department of Radiology, School of Medicine, University of Yamanashi, Chuo, Yamanashi, 409-3898, Japan; Department of Radiology, School of Medicine, University of Yamanashi, Chuo, Yamanashi, 409-3898, Japan; Department of Radiology, Shizuoka General Hospital, Shizuoka, Shizuoka, 420-8527, Japan; Department of Radiology, School of Medicine, University of Yamanashi, Chuo, Yamanashi, 409-3898, Japan; Department of Radiology, School of Medicine, University of Yamanashi, Chuo, Yamanashi, 409-3898, Japan; Proton Therapy Center, Aizawa Hospital, Matsumoto, Nagano, 390-8510, Japan; Department of Radiology, Toranomon Hospital, Minato, Tokyo, 105-8470, Japan; Department of Radiology, Shimada Municipal Hospital, Shimada, Shizuoka, 427-8502, Japan; Department of Radiology, School of Medicine, University of Yamanashi, Chuo, Yamanashi, 409-3898, Japan; Department of Radiology, Yamanashi Prefectural Hospital, Yamanashi, Yamanashi, 400-8506, Japan; Department of Radiology, School of Medicine, University of Yamanashi, Chuo, Yamanashi, 409-3898, Japan

**Keywords:** modified Glasgow prognostic score (mGPS), non-small cell lung cancer (NSCLC), stereotactic body radiation therapy (SBRT), biological markers, prognosis

## Abstract

We aimed to assess the predictive value of the modified Glasgow prognostic score (mGPS) in patients with non-small cell lung cancer (NSCLC) who underwent stereotactic body radiation therapy (SBRT). We retrospectively reviewed the records of 207 patients, with a median age of 79 years. The pretreatment mGPS was calculated and categorized as high (mGPS = 1–2) or low (mGPS = 0). The median follow-up duration was 40.7 months. The five-year overall survival (OS), progression-free survival (PFS) and time to progression (TTP) rates were 44.3%, 36% and 54.4%, respectively. Multivariate analysis revealed that mGPS was independently predictive of OS (hazard ratio [HR] 1.67; 95% confidence interval 1.14–2.44: *P* = 0.009), PFS (HR 1.58; 1.10–2.28: *P* = 0.014) and TTP (HR 1.66; 1.03–2.68: *P* = 0.039). Patients who had high mGPS showed significantly worse OS (33.3 vs 64.5 months, *P* = 0.003) and worse PFS (23.8 vs 39 months, *P* = 0.008) than those who had low mGPS. The data showed a trend that patients with high mGPS suffered earlier progression compared to those with low mGPS (54.3 vs 88.1 months, *P* = 0.149). We confirmed that mGPS is independently predictive of prognosis in NSCLC patients treated with SBRT.

## INTRODUCTION

Lung cancer is the second most common cancer and is the leading cause of cancer deaths globally [1, 2]. Non-small cell lung cancer (NSCLC) accounts for 80–85% of newly diagnosed lung cancers [3]. Stereotactic body radiation therapy (SBRT) has become the best alternative to surgery for early-stage, medically inoperable NSCLC patients and provides excellent survival and local control outcomes [4, 5]. However, regional and distant failures occur in 30% of patients, which remains a problem [6]. Early diagnosis and intervention for recurrence or metastasis may reduce lung cancer mortality. Therefore, an easily measurable prognostic tool is necessary.

Recently, there has been increasing evidence showing that inflammatory and nutritional states have prognostic value in patients of NSCLC [7, 8]. The serum C-reactive protein (CRP) and albumin levels are good indicators of systemic inflammation and nutritional status [9, 10]. The modified Glasgow prognostic score (mGPS), a representative maker based on CRP and albumin, has been shown to be a prognostic indicator for various types of cancer, including lung cancer [11–14]. However, most reports regarding the mGPS have focused on patients treated with surgery or chemotherapy [15, 16]. Kishi *et al.* published the first report on the prognostic value of mGPS in NSCLC patients treated with SBRT [17]. However, the predictive value of the mGPS has not yet been fully addressed. This study aimed to evaluate whether the mGPS had multivariable-adjusted prognostic value for clinical outcomes among patients with NSCLC receiving SBRT.

## METHODS AND MATERIALS

### Ethics

This study conformed to the Ethical Principles for Medical Research Involving Human Subjects that were issued by the Ministry of Health, Labour and Welfare and the Ministry of Education, Culture, Sports, Science, and Technology in Japan. The retrospective study protocol was approved by the institutional review board of University of Yamanashi (#1582).

### Patients

We retrospectively reviewed the records of NSCLC patients who underwent SBRT at our institution from 2001–2016. The inclusion criteria were: (i) pathologically confirmed stage I primary NSCLC based on the seventh TNM classification [18], (ii) detailed medical records were available, and (iii) laboratory test results were available starting from < 3 months before SBRT. The exclusion criteria were: (i) the presence of other active primary cancers, (ii) received pretreatment surgery before the SBRT, and (iii) received concurrent or consecutive chemotherapy. The pretreatment work-up included taking a complete medical history-taking, physical examination, pulmonary function tests, chest radiography, computed tomography (CT), magnetic resonance imaging of the brain and whole-body ^18^F-fluorodeoxyglucose (FDG) positron emission tomography (PET)/CT.

### Treatments

All patients were irradiated using a linear accelerator using multiple noncoplanar static ports or dynamic arcs. Our SBRT method for NSCLC has been described in detail previously [19]. Imaging guidance was based on kilovoltage CT images acquired by an in-room CT system. The self-held breath-hold technique using a respiratory monitoring device was used for respiratory management [20]. The therapeutic strategy gradually changed during the long study period. Before 2005, the isocenter was prescribed a dose of 60 Gy in 10 fractions for T1 lesions or a dose of 70 Gy in 10 fractions for T2 lesions. During 2005–2010, both T1 and T2 lesions were prescribed at the dose covering 95% of the volume (D95) of the planning target volume (PTV) receiving 48 Gy in 4 fractions. After 2010, the prescribed dose was increased in an attempt to achieve better local control to 50 Gy for T1 lesions or 55 Gy for T2 lesions in 4 fractions to the PTV-D95. If the tumor was located close to an organ at risk, 60 or 70 Gy in 10 fractions to the PTV-D95 was administered to meet the dose constraints, regardless of the size of the primary tumor.

### Follow-up

A follow-up CT was scheduled every three months during the first year, every three to six months during the second year, and then every six to 12 months for at least five years. When the CT revealed abnormal findings, FDG-PET/CT was recommended. Recurrence was diagnosed clinically or pathologically. When recurrence was diagnosed clinically based on abnormal imaging findings, serum tumor markers, or physical findings, the recurrence date was defined as the date of detection of the findings that indicated recurrence. When recurrence was diagnosed pathologically, the recurrence date was defined as the date of collection of the tissue or cell specimen.

### Modified Glasgow prognostic score

The original Glasgow prognostic score (GPS) is a 4-point scale that was developed to predict the prognoses of patients receiving chemotherapy for advanced NSCLC [21]. For scoring, one point is added for each of the following criteria: stage IV disease, performance status (PS) score of 2–4, CRP levels > 1.0 mg/dL and albumin levels < 3.5 g/dL. The mGPS was developed from the GPS and simplified it by omitting stage and PS. Therefore, the maximum score of mGPS is only 2. Due to technological advancements, the threshold levels of mGPS were slightly changed [22]. We calculated the mGPS based on a cut-off value of 0.3 mg/dL for CRP and 3.5 mg/dL for albumin to facilitate a comparison between our results and those of previous studies (Table 1). The mGPS was calculated using the most recent laboratory data, which were obtained < 3 months before the first day of SBRT.

**Table 1:** Definition of the modified Glasgow prognostic score

mGPS	Description
0	CRP < 0.3 mg/dL and ALB > 3.5 mg/dL
1	CRP < 0.3 mg/dL and ALB ≤ 3.5 mg/dL
1	CRP ≥0.3 mg/dL and ALB > 3.5 mg/dL
2	CRP ≥0.3 mg/dL and ALB ≤ 3.5 mg/dL

mGPS, modified Glasgow prognostic score; CRP, C-reactive protein; ALB, albumin

### Survival-related outcomes

The primary outcome of interest was overall survival (OS), which was defined as the time from the start of SBRT to death from any cause. The secondary outcomes were progression-free survival (PFS) and time to progression (TTP). The PFS interval was defined as the time from the start of SBRT to the first instance of tumor progression or death from any cause. The TTP was defined as the time from the start of SBRT to the first appearance of local recurrence or regional/distant metastasis (DM).

### Statistical analysis

The chi-squared test was used to compare clinicopathological parameters between mGPS groups. Survival curves were estimated using the Kaplan–Meier method and compared using the log-rank test. The associations between the survival outcomes and tumor characteristics were assessed using univariate and multivariate Cox proportional hazards models. The potential prognostic factors were age, sex, PS, T-stage, histological subtype and mGPS [17]. Differences were considered statistically significant at *P*-values of < 0.05, and all analyses were performed using IBM SPSS Statistics for Macintosh software (version 22.0; IBM Corp., Armonk, NY).

## RESULTS

### Patient characteristics

From an initial 328 patients who underwent SBRT in this period, 121 patients were excluded for the following reasons: 31 had multiple cancers detected; eight underwent consecutive chemotherapy or irradiation; 54 were deleted for lack of histological information; and 28 had no laboratory data available for the mGPS. Therefore, 207 patients were included in this study. All patients were medically inoperable or had refused surgery. The prescribed doses were 48 Gy in 4 fractions (35.7%), 50 Gy in 4 fractions (18.4%), 60 Gy in 10 fractions (14.5%), 70 Gy in 10 fractions (13%) and 55 Gy in 4 fractions (13%). The median biologically effective dose was 105.6 Gy (range, 80–150 Gy) based on an alpha/beta value = 10.

As shown in Table 2, this cohort contains 149 men and 58 women, with a median age of 79 years (range: 53–91 years). Most patients had a PS score of 0 (93.7%). The T-stage was T1a in 69 patients (33.3%), T1b in 56 patients (27.1%) and T2a in 82 patients (39.6%). Histological examination showed adenocarcinoma (Ad) in 124 patients (59.9%), squamous cell carcinoma (SCC) in 59 patients (28.5%), and non-Ad/non-SCC in 24 patients (11.6%). Table 2 also compares the clinicopathological parameters between the groups. Sex (*P* = 0.01), T-stage (*P* = 0.041) and histological subtype (*P* = 0.015) were significantly different between the two groups.

**Table 2:** Relationships between the mGPS and various clinicopathological parameters

		All patients n = 207	mGPS 0 n = 136	mGPS 1–2 n = 71	p-value
Age, years	Median: 79 years				
	<75	51 (24.6)	37 (27.2)	14 (19.7)	0.235
	≥75	156 (75.4)	99 (72.8)	57 (80.3)	
Sex	Female	58 (28.0)	46 (33.8)	12 (16.9)	0.010
	Male	149 (72.0)	90 (66.2)	59 (83.1)	
ECOG-PS	0–1	194 (93.7)	129 (94.9)	65 (91.5)	0.352
	≥2	13 (6.3)	7 (5.1)	6 (8.5)	
T-stage	T1a	69 (33.3)	45 (33.1)	24 (33.8)	0.041
	T1b	56 (27.1)	30 (22.0)	26 (36.6)	
	T2a	82 (39.6)	61 (44.9)	21 (29.6)	
Histological subtype	Adenocarcinoma	124 (59.9)	90 (66.2)	34 (47.9)	0.015
	SCC	59 (28.5)	30 (22.0)	29 (40.8)	
	Other	24 (11.6)	16 (11.7)	8 (11.3)	

mGPS, modified Glasgow prognostic score; ECOG-PS, Eastern Cooperative Oncology Group performance status; SCC, squamous cell carcinoma

Data are shown as numbers (%)

### Modified Glasgow prognostic score

The median interval from blood collection to the first day of SBRT was six days (range: 0–90 days; interquartile range: 4–11 days). The mean (standard deviation) pretreatment values were 0.45 (0.97) mg/dL for CRP and 3.90 (0.44) mg/dL for albumin. In this cohort of patients, the mGPS values were 0 for 136 patients (65.7%), 1 for 52 patients (25.1%) and 2 for 19 patients (9.2%). Incidentally, they had the same GPS and mGPS value (Stage < 4 = 0 point; PS < 2 = 0 point). An mGPS of 2 was rare, this is contrast to scores from patients receiving or about to receive chemotherapy. A previous study combined patients with an mGPS of 1 and 2 into a single group for analyses [17]. To facilitate the comparison of our findings, we also combined these two groups. Therefore, the patients were categorized into two groups: 136 patients with a low mGPS (mGPS = 0) and 71 patients with a high mGPS (mGPS = 1–2).

### Relationships between the mGPS and survival-related outcomes

**Fig. 1.**
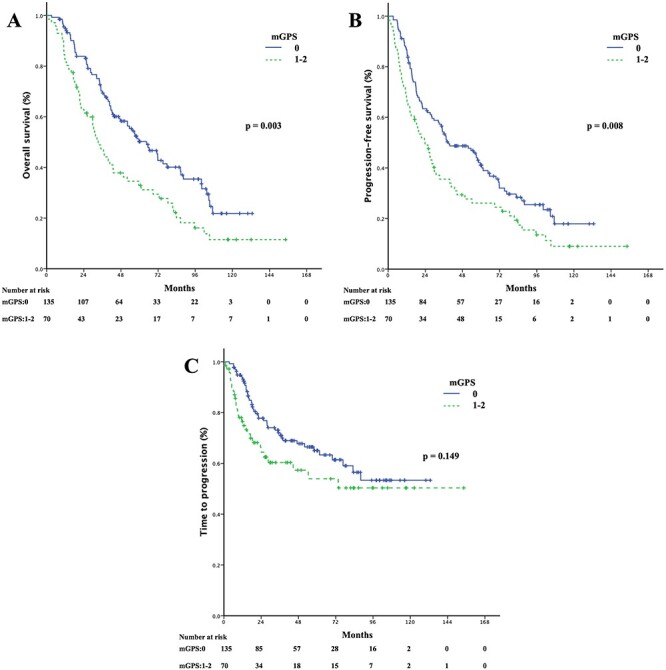
Kaplan–Meier curves for: (i) overall survival, (ii) progression-free survival, and (iii) time to progression according to the pretreatment modified Glasgow prognostic score (mGPS, 0 vs 1–2).

The median follow-up duration was 40.7 months (range: 1–154.3 months). The estimated median time of OS, PFS and TTP were 52.2 months (95% confidence interval [CI]: 39.6–64.8 months), 33.3 months (95% CI: 25.7–40.9 months) and 76.7 months (95% CI: not estimated), respectively. Patients who had a high mGPS had showed significantly worse OS (33.3 vs 64.5 months, *P* = 0.003) and worse PFS (23.8 vs 39 months, *P* = 0.008) than those who had a low mGPS. Although, no significant difference was found between the subgroups in TTP (54.3 vs 88.1 months, *P* = 0.149), the data showed a strong trend that patients with a high mGPS suffered an early progression in about 2.8 years. The three-year rates were 61.8% for OS, 47.8% for PFS and 76.7% for TTP. The five-year rates were 44.3% for OS, 36% for PFS and 54.4% for TTP (Table 3). The curves for survival-related outcomes are shown in Fig. 1. In total, 142 patients (68.6%) died. Of these, 47 (22.7%) died of primary lung cancer, 92 (44.4%) died of other diseases or unknown causes and three (1.4%) died of treatment-related toxicities. Among the 150 patients (72.5%) who had disease progression, 43 (20.8%) developed local recurrence, 35 (16.9%) developed regional lymph nodes metastasis and 72 (34.8%) developed DM.

**Table 3:** Survival outcomes

	All patients	mGPS 0	mGPS 1–2
Overall survival			
Median (months)	52.2	64.5	33.3
Three-year rate (%)	61.8	69.4	45.3
Five-year rate (%)	44.3	50.2	32.9
Progression-free survival			
Median (months)	33.3	39.0	23.8
Three-year rate (%)	47.8	54.2	35.5
Five-year rate (%)	36.0	41.1	26.1
Time to progression			
Median (months)	76.7	88.1	54.3
Three-year rate (%)	61.1	64.8	53.5
Five-year rate (%)	54.4	57.6	47.8

mGPS, modified Glasgow prognostic score

### Prognostic performance of pretreatment mGPS

The results of the univariate and multivariate analysis are given in Table 4. In the univariate analysis, the mGPS (hazard ratio [HR]: 1.67; 95% CI: 1.19–2.34; *P* = 0.003), sex (HR: 2.29; 95% CI: 1.51–3.47; *P* < 0.001), PS (HR: 2.24; 95% CI: 1.23–4.06; *P* = 0.006), and histological subtype (SCC vs Ad, HR: 1.53; 95% CI: 1.07–2.10; non-Ad/non-SCC vs Ad, HR: 0.95; 95% CI: 0.55–1.65; *P* = 0.049) were significantly associated with OS. The mGPS (HR: 1.54; 95% CI: 1.12–2.13; *P* = 0.008) and sex (HR: 1.88; 95% CI: 1.28–2.75; *P* = 0.001) were significantly associated with PFS. No significant difference could be observed in the TTP. However, the Cox regression model suggested an association with an HR of 1.38 (95% CI: 0.89–2.13).

**Table 4:** Univariate and multivariate Cox regression analyses of overall survival, progression-free survival, and time to progression

		**Overall survival**				**Progression-free survival**				**Time to progression**			
		Univariate analysis		Multivariate analysis		Univariate analysis		Multivariate analysis		Univariate analysis		Multivariate analysis	
		HR (95% CI)	p-value	HR (95% CI)	p-value	HR (95% CI)	p-value	HR (95% CI)	p-value	HR (95% CI)	p-value	HR (95% CI)	p-value
**Age**	<75	1	0.669	1	0.956	1	0.956	1	0.704	1	0.507	1	0.295
	≥75	1.09 (0.74–1.61)		0.99 (0.64–1.53)		1.01 (0.70–1.46)		0.92 (0.61–1.39)		0.85 (0.53–1.37)		0.76 (0.45–1.28)	
**Sex**	Female	1	<0.001^*^	1	0.001^*^	1	0.001^*^	1	0.010^*^	1	0.434	1	0.660
	Male	2.29 (1.51–3.47)		2.22 (1.11–3.50)		1.88 (1.28–2.75)		1.75 (1.14–2.67)		1.20 (0.76–1.90)		1.12 (0.67–1.89)	
**ECOG-PS**	0–1	1	0.006^*^	1	0.008^*^	1	0.061	1	0.076	1	0.740	1	0.671
	≥2	2.24 (1.23–4.06)		2.31 (1.25–4.27)		1.75 (0.97–3.16)		1.73 (0.94–3.19)		1.17 (0.47–2.88)		1.22 (0.49–3.07)	
**T-stage**	T1a	1	0.395	1	0.198	1	0.165	1	0.065	1	0.125	1	0.051
	T1b	1.12 (0.74–1.71)		0.98 (0.66–1.54)		1.00 (0.66–1.51)		0.92 (0.59–1.42)		0.95 (0.53–1.69)		0.93 (0.51–1.69)	
	T2a	1.32 (0.88–1.95)		1.38 (0.91–2.09)		1.36 (0.94–1.98)		1.43 (0.97–2.10)		1.51 (0.93–2.46)		1.66 (1.00–2.76)	
**Pathology**	Adenocarcinoma	1	0.049	1	0.446	1	0.236	1	0.693	1	0.980	1	0.844
	SCC	1.53 (1.07–2.10)		0.93 (0.62–1.40)		1.33 (0.94–1.89)		0.92 (0.63–1.36)		1.07 (0.55–2.10)		0.85 (0.49–1.47)	
	Other	0.95 (0.55–1.65)		0.69 (0.38–1.23)		0.95 (0.59–1.66)		0.79 (0.46–1.37)		1.05 (0.50–2.20)		0.93 (0.46–1.90)	
**mGPS**	0	1	0.003^*^	1	0.009^*^	1	0.008^*^	1	0.014^*^	1	0.149	1	0.039^*^
	1–2	1.67 (1.19–2.34)		1.67 (1.14–2.44)		1.54 (1.12–2.13)		1.58 (1.10–2.28)		1.38 (0.89–2.13)		1.66 (1.03–2.68)	

HR, hazard ratio; CI, confidence interval; ECOG-PS, Eastern Cooperative Oncology Group performance status; SCC, squamous cell carcinoma; mGPS, modified Glasgow prognostic score

^*^Statistically significant

In multivariate analysis, the mGPS (HR: 1.67; 95% CI: 1.14–2.44; *P* = 0.009) was significantly associated with OS, along with sex (HR: 2.22; 95% CI: 1.11–3.50; *P* = 0.001) and PS (HR: 2.31; 95% CI: 1.25–4.27; *P* = 0.008). The mGPS (HR: 1.58; 95% CI: 1.10–2.28; *P* = 0.014) and sex (HR: 1.75; 95% CI: 1.14–2.67; *P* = 0.010) were significantly associated with PFS. After adjustment for clinical cofactors, only the mGPS (HR: 1.66; 95% CI: 1.03–2.68; *P* = 0.039) was significantly associated with TTP.

## DISCUSSION

In this study, we showed that the mGPS is significantly associated with survival in a univariate analysis. After adjusting for clinical confounders, the mGPS was the only factor associated with both survival and cancer progression. The results from the present study indicate that the mGPS may serve as an independent prognostic factor in NSCLC patients treated with SBRT. To the best of our knowledge, this study evaluated the largest number of cases for the relationship between the mGPS and survival-related outcomes in NSCLC patients treated with SBRT.

Limited studies have been done to assess the prognostic value of the mGPS in NSCLC patients treated with SBRT. The original GPS was introduced in 2003 to predict the prognosis of patients receiving chemotherapy for advanced NSCLC [21]. In 2011, Proctor *et al*. [23] reported that a simple combination of only CRP and albumin (the mGPS) showed independent prognostic value in cases of lung cancer, they subsequently reported that the mGPS was superior to the GPS [24]. In 2017, Jin *et al.* [25] performed a meta-analysis, which showed that a high mGPS was significantly associated with poor OS (HR: 1.77, 95% CI: 1.35–2.31; *P* < 0.05). However, most studies assessed the prognostic value of mGPS in patients treated with chemotherapy or surgery. Kishi *et al.* [17] reported for the first time the clinical utility of the mGPS in NSCLC patients treated with SBRT. They reported that a high mGPS was significantly correlated with lung cancer mortality (HR: 1.82, 95% CI: 1.24–2.26; *P* = 0.002). This is consistent with our findings, which had an HR of 1.67 (95% CI: 1.19–2.34) was obtained. They performed a univariate Gray’s test and found that patients with a high mGPS were more likely to experience DM. They suggested that a reduction of DM was a way to improve outcomes. However, little evidence about local and regional recurrence is available. Our results show that a high mGPS might serve as a prognostic factor to detect patients who could suffer from early disease progression. Kishi *et al.* [17] also reported that female patients had significantly better OS than male patients, which agrees with our findings, in which male patients had an approximately 2-fold higher risk of worse OS and worse PFS. Furthermore, we found that PS was significantly associated with the OS in a univariate analysis. However, it only showed a suggestive association with the survival after multivariable adjustment. T stage is commonly applied to survival prediction for patients who undergo SBRT for NSCLC [26], but our results did not support this.

Previous reports have shown that inflammation is recognized as a very important part of tumorigenesis and cancer progression, especially lung cancer [27–29]. Recent studies have elucidated the mechanisms by which systemic inflammation negatively influences survival. Inflammation can be triggered by infectious or non-infectious agents [30]. The release of pro-inflammatory cytokines, including interleukin-1 (IL-1) and tumor necrosis factor-alpha (TNF-α), plays a key role in dissociation of nuclear factor kappa B (NF-kB) from its inhibitors. NF-kB is able to induce an increase in IL-6 production, which results in the release of acute-phase reactants including CRP [31, 32]. Conversely, albumin levels are reduced during chronic inflammation owing to increased vascular permeability and decreased hepatic albumin synthesis [10, 33]. There is also evidence that serum albumin concentrations can serve as a valuable predictor of nutritional status in cancer patients, and malnutrition is correlated with poor survival [33, 34]. Our findings suggest that the mGPS, which is a combination of serum CRP and albumin levels, might serve as an indicator of chronic inflammation and malnutrition, which result in a worse prognosis. Patients with a high pretreatment mGPS might benefit from inhibiting the systemic inflammatory reaction and/or nutritional intervention.

SBRT is a common treatment option for medically inoperable patients. In a matched pairs analysis, Matsuo *et al.* [35] found that the difference in OS was not significant between SBRT and surgery at five years (40.4% vs 55.6%; *P* = 0.124), and cause specific survival was comparable between the groups (35.3% vs 30.3%; *P* = 0.427). However, high rates of regional and distant failure remain a problem for SBRT. Early cancer progression always links to worse survival. There are many studies that evaluated prognostic factors in order to identify high-risk patients. Using this information, clinical practitioners can prioritize adjuvant therapy for those patients in order to improve survival. However, there are no studies on the relationship between these prognostic factors and relapse time. Therefore, we analyzed the relationship between mGPS and TTP, and we found that patients with a high mGPS had faster disease progression than those who had a low mGPS. The mGPS can provides clinical practitioners with a quantitative model for finding patients at risk and individualizing treatment planning. We recommended that patients who have a high mGPS should be considered for adjuvant intensive systemic therapies, if tolerable. A combination of SBRT and immunotherapy seems to be a promising option, which has shown positive results in locally advanced and metastatic NSCLC [36, 37]. Immunotherapy is tolerated better than chemotherapy in medically inoperable patients, who usually cannot receive chemotherapy to help with distant control [38]. A series of studies have confirmed the short-term safety of combining SBRT and immunotherapy [39–42]. However, there is no clear consensus available on the optimal radiation dose and schedule for combining SBRT with immunotherapy [43]. We hypothesize that use of immunotherapy will reduce locoregional and distant recurrence with a resulting improvement in OS following SBRT for NSCLC. Further investigations are warranted.

Our study has some limitations. First, the single-institution retrospective design is prone to selection bias. Second, treatment strategies have changed during the study period (2001–2016). Although these strategies may be confounding factors in predicting cancer prognoses, we did not have enough information to assess them. Third, evaluation of the TTP as an end-point may have been influenced by the diagnostic accuracy of disease progression and too many cases being censored. Fourth, the accuracy and precision of CRP and albumin measurement also changed during the study period. Thus, further studies are needed to address these issues and confirm whether the mGPS is a useful clinical prognostic factor for patients planning to undergo SBRT for NSCLC.

In conclusion, our results indicated that the mGPS is predictive of the prognosis of NSCLC patients undergoing SBRT after multivariable adjustment, with higher values predicting worse survival outcomes. The mGPS can be calculated easily and quickly using routinely available and inexpensive tests, making it a valuable clinical tool.

## CONFLICT OF INTEREST

The authors declare that there are no conflicts of interest.

## PREVIOUS PRESENTATION

Portions of this work were presented at the 61st annual meeting of the American Society for Radiation Oncology (ASTRO), September 15–18, 2019, Chicago, IL.

## IRB NUMBER

University of Yamanashi Institutional Review Board approval, No. 1582 (Feb 16, 2017)

## Notes

**Statistical Analysis Author**: Dr. Eiji Nakatani, Division of Statistical Analysis, Research Support Center, Shizuoka General Hospital, 4-27-1 Kita-Ando, Shizuoka City, Shizuoka, 420-8527, JAPAN. Tel.: +81-54-247-6111, Fax: +81-54-247-6140, Email: nakatani.eiji.int@gmail.com
